# Experimental study on anisotropic unloading mechanical behavior of bedded sandstone

**DOI:** 10.1038/s41598-023-41073-w

**Published:** 2023-08-26

**Authors:** Jingcheng Fang, Huafeng Deng, Wei Wang, Jianlin Li, Eleyas Assefa

**Affiliations:** 1 Key Laboratory of Geological Hazards on Three Gorges Reservoir Area (China Three Gorges University), Ministry of Education, Yichang, 443002 Hubei China; 2 Transportation Intelligence Center of Yichang, Yichang, 443000 Hubei China; 3 College of Engineering, Addis Ababa Science and Technology University, Addis Ababa, Ethiopia

**Keywords:** Engineering, Civil engineering

## Abstract

Sandstone is a kind of bedded rock mass commonly used in engineering. The deformation and strength of bedded sandstone impose some problems during excavation. In this study, triaxial unloading tests were conducted on rock specimens (considering seven different bedding angles (*β*)). The results revealed the following key points: (1) At a constant confining pressure, the elastic modulus was gradually increasing when the bedding angle increased. Furthermore, after initial decreasing, the deformation modulus was increased and had a U-shaped distribution. (2) During the unloading of the axial compression, the rate of axial strain variations was initially increased and then decreased while the bedding angle increased (it has exhibited an inverted U-shaped distribution). However, the peak strength, cohesion, and angle of internal friction of rock specimens showed an initial decreasing and then increasing trend. (3) During the loading and unloading stages, the confining pressure reduces the anisotropy of bedded rock masses. (4) In the triaxial unloading test, the failure of rock specimens can be classified into four modes. When there was a large intersection angle between the bedding plane and the unloading direction, failure developed at the bedding planes.

## Introduction

A bedded rock mass is common in engineering construction. It exhibits different deformation and strength characteristics at parallel, perpendicular, or oblique bedding planes. This is because bedding planes have different rock matrix and physical and mechanical properties. Bedded rock mass is a typical transversely isotropic body, and transversely isotropic body is a special case of isotropic body. The elastic properties of all directions in a plane of rock are the same, and this plane is called isotropic plane, but the mechanical properties perpendicular to this plane are different, and objects with this property are called transversely isotropic bodies. The characteristic of transversely isotropic body is that it has the same elasticity parallel to the isotropic plane (transversely). Due to the transverse isotropic nature of the laminated rock mass, the strength of the rock mass is not only related to the strength of the intact rock itself, but also related to the mechanical properties of the structural face. Under different stress conditions, the damage surface will be affected by the angle between the principal stress and the structural surface, and the damage surface will be along the structural surface or through the rock, thus exhibiting different mechanical properties. The anisotropic nature of bedded rock mass poses a significant challenge in design and construction.

They have very noticeable deformation and strength anisotropy at different bedding angles. Many scholars investigated the anisotropic mechanical properties of bedded rock through uniaxial or triaxial compression tests. The uniaxial compression strength tests of rocks at different bedding angles exhibited anisotropic strength and elastic properties^1–5^. The loading angle (The angle between the loading direction and the weak bedding plane) and confining pressure govern the Young’s modulus and the tensile strength of bedded sandstone^6–10^. The mechanical behavior and energy release characteristics of layered sandstone with different azimuth bedding have obvious anisotropy under uniaxial compression^11^. Moreover, specimens with different bedding angles and thicknesses exhibited anisotropic mechanical characteristics under direct shear, uniaxial and triaxial tests^12^. The orientation of the plane of anisotropy (bedding or cleavage plane) and the direction of diametral loading plays a leading role in the mechanical behavior of anisotropic sedimentary rocks^13–15^.

The findings of this paper play a guiding role in understanding the anisotropic mechanical properties of bedded rock masses. However, practicing engineers often encounter many complicated problems during rock mass excavation, such as high and steep slopes or underground chambers and foundation excavations. Moreover, there are fundamental variations in the mechanical properties of rock mass during loading and unloading conditions^16,17^. Based on stability analysis of high slope excavation on the Three Gorges permanent ship lock, the concept of unloading rock mechanics has been developed and gradually acknowledged^18–21^. The true triaxial unloading test showed that the strength and failure modes of cubic rock specimens were affected by intermediate principal stress^22–26^. However, during the triaxial test (with different loading and unloading paths), the deformation and damage extent of granite specimens can be described by taking the ratio of dissipated strain energy to the total strain energy^27^. Some studies reported that the confining pressure increases the shear stress and the shear strength of the fracture surface during the unloading stage, which increases the possibility of sliding shear^28^. Based on the principle of energy, the absorbed axial strain energy (at different unloading paths) is mainly converted to the axial compressive energy. Besides, the impact of initial confining pressure on the strain energy was more significant than that of the unloading path^29^.

Some scholars have also studied the anisotropic nature of unloading rock mechanics. At the same confining pressure, a vertical-bedding plane has higher strength than horizontal bedding^30^. The triaxial unloading test on a bedded marble showed that the deformation on a horizontal layer is higher than the vertical one, and it was anisotropic^31^. Anisotropic coefficients describe the softening during unloading, and a transversely isotropic rock constitutive model was established based on the unloading characteristics of mica-quartz schist^32^. The rock salt underwent expansion under constant axial compression and confining pressure^33^.

To sum up, many scholars have studied the anisotropic mechanical properties of bedded rock mass by uniaxial compression or triaxial compression tests and those studies have laid a good foundation for understanding the anisotropic mechanical properties of the bedded rock mass. However, there are still some gaps. In the process of engineering construction, a large number of complex rock mass excavation problems are often encountered, such as high and steep slope, underground cavern, foundation excavation and so on. Rock mass excavation is an unloading process. At present, in the research on the anisotropic unloading mechanical properties of bedded rock mass, only parallel and vertical bedding are usually considered, and there is no related research on the influence of different bedding angles on the unloading mechanical properties of bedded rock mass. Therefore, further study is needed.

Sandstone is a typical sedimentary rock (that is widely available in many engineering projects). Due to the complex nature of the excavation, there will be transformations between the unloading direction and bedding. Accordingly, the corresponding unloading mechanical characteristics are different. If it is not properly addressed during design and construction, it will create problems on safety issues. Therefore, this paper studied the anisotropic unloading mechanical properties of sandstone (with different bedding angles) through a triaxial unloading test.

## Methods and materials

### Rock sample preparation

**Figure 1:**
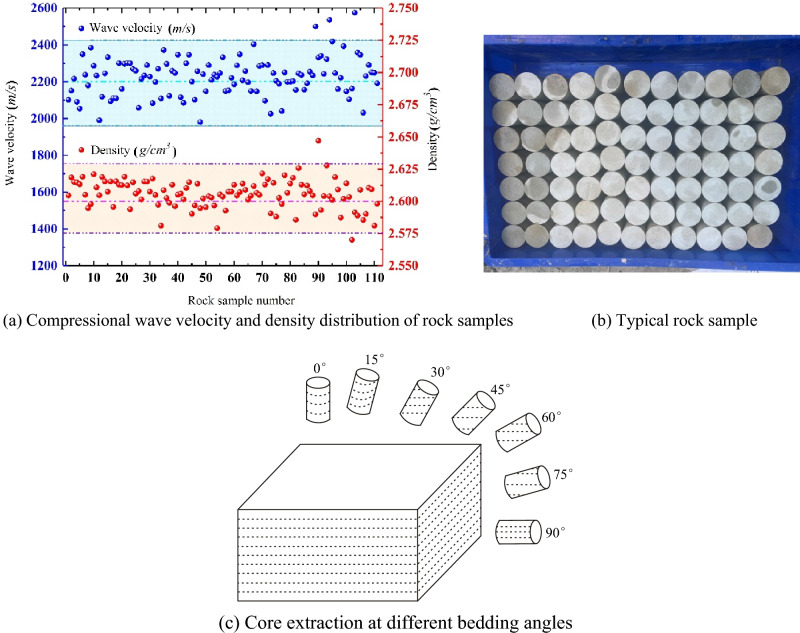
Compressional wave velocity and density distribution of rock samples.

The rock samples involved in this experimental study are taken from the Tanjiahe landslide in the Three Gorges Reservoir area, which is a bedding bedrock landslide, which involves a wide range of rock mass excavation in the process of highway construction in the reservoir area. And the rock is Jurassic quartz sandstone, which are pore-type calcareous cemented sericite medium-grained quartz sandstone, slightly weathered, with good integrity, whose grain size is about 0.3 to 0.5 mm. The standard cylindrical rock samples of Φ 50 mm × L100 mm (Φ is the diameter, L is the height) were processed in strict accordance with the specification, and then strictly screened according to the compressional wave velocity and density^34,35^, and the results of compressional wave velocity and density of the measured rock samples are shown in Fig. 1a, and the typical rock samples screened are shown in Fig. 1b. Among them, the density range of rock samples is about 2.57–2.65 g/m^3^, and the average value is about 2.60 g/m^3^; the compressional wave velocity range of rock samples is about 2234–2430 m/s, and the average value is about 2200 m/s. Except for individual rock samples with slightly large fluctuations in compressional wave velocity and density, most of the compressional wave velocity and density of rock samples are more concentrated, which indicates that the dispersion of rock samples is small, and the rock samples with more concentrated compressional wave velocity and density are selected for the test. The conceptual diagram for different bedding angles is shown in Fig. 1c.

**Figure 2:**
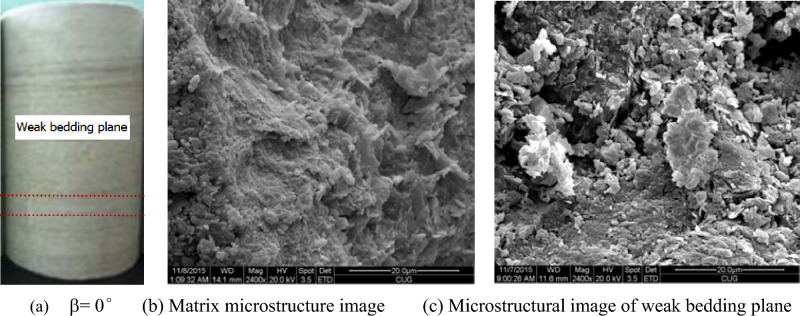
Weak bedding plane and microstructural images.

As shown in Fig. 2a, the weak plane of bedding is obvious from the side of the sample. In order to observe the characteristics and differences of microstructure of matrix and bedding weak surface of layered sandstone, scanning electron microscope analysis was carried out. The mineral particles of sandstone matrix structure are closely arranged, pores and fractures are few, while micro pores and fissures of bedding weak surface are obviously developed, as shown in Fig. 2b,c.

### Experimental instruments

**Figure 3:**
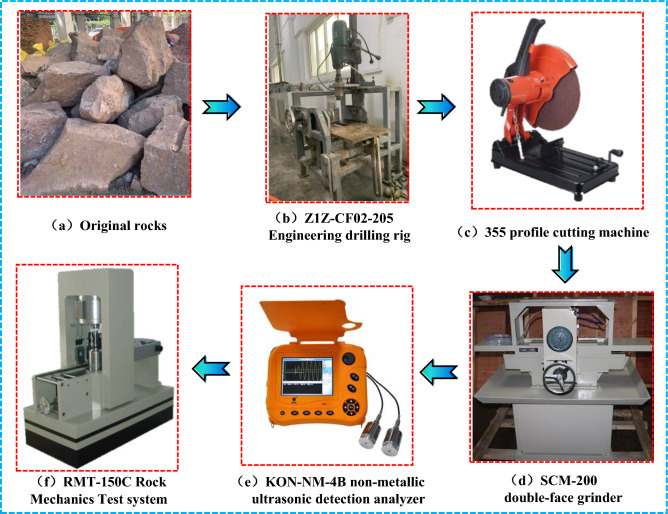
Experimental equipment and experimental flow chart.

The experimental program involved five steps. Namely: rock drilling, cutting, grinding, screening, and testing. Specific instruments are illustrated in Fig. 3.

50 mm rock cores were extracted by using the Z1Z-CF02-205 drilling device (Fig. 3b). Several cylindrical rock specimens (with a 100 mm height) were prepared using a cutting machine (Fig. 3c). Cylindrical samples were polished using SCM-200 double-face grinder (Fig. 3d) to meet the standard precision requirements. So that the variation in the height and diameter of the rock sample was not greater than 0.3 mm, the roughness at the top and bottom of the specimen was not greater than 0.05 mm, and the deviation between the end face and the axis was less than 0.25°. The KON-NM-4B non-metallic ultrasonic testing analyzer (Fig. 3e) was used to screen noticeable discreteness among polished rock samples based on their compressional wave velocity. The RMT-150C rock mechanics test system (Fig. 3f) was installed with displacement and force sensors, which can measure axial deformation, circumferential deformation, and load in real-time; meanwhile, the equipment has good dynamic and static characteristics and system stiffness, which can track the instantaneous damage of rocks and make satisfactory stress–strain curves for a variety of rocks, and the test equipment can keep the test load stable for a long time to ensure the normal behavior of the test. Hence, mechanical tests were conducted on the selected samples using this device.

### Experimental design

**Figure 4:**
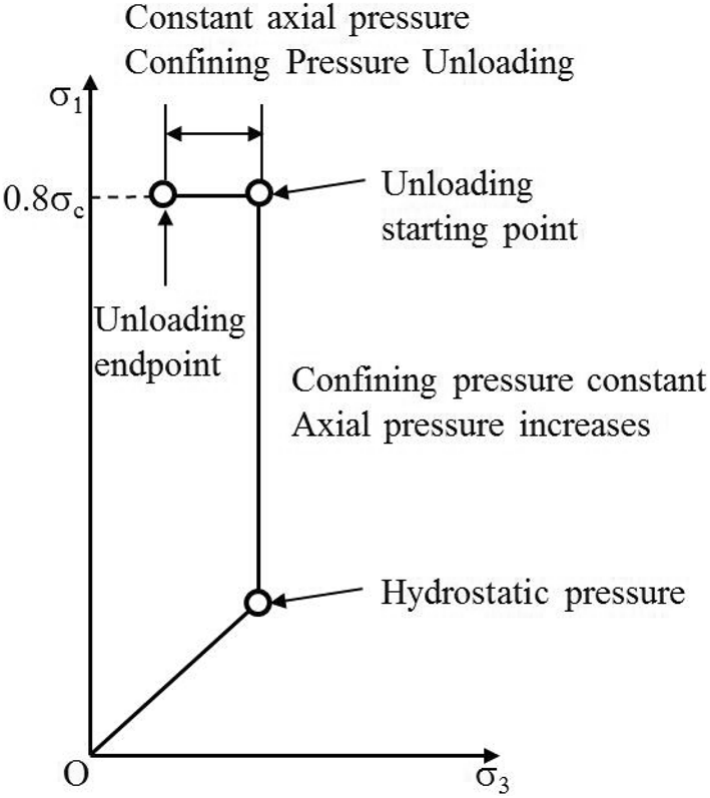
Stress path.

The stress path is shown in Fig. 4. The triaxial compressive strength of rock specimens was determined at different confining pressures (5, 10, 15, and 20 MPa). In due course, the confining pressure was unloaded while the axial compression was kept constant. Initially, the confining pressure was set to the corresponding hydrostatic pressure, and then the axial compression was increased to 80% of the compressive strength at a rate of 0.5 MPa/s. Finally, the axial stress was kept constant, and the confining pressure was gradually reduced until failure (at a rate of 0.02 MPa/s).

## Results

### Anisotropic stress–strain curve

**Figure 5:**
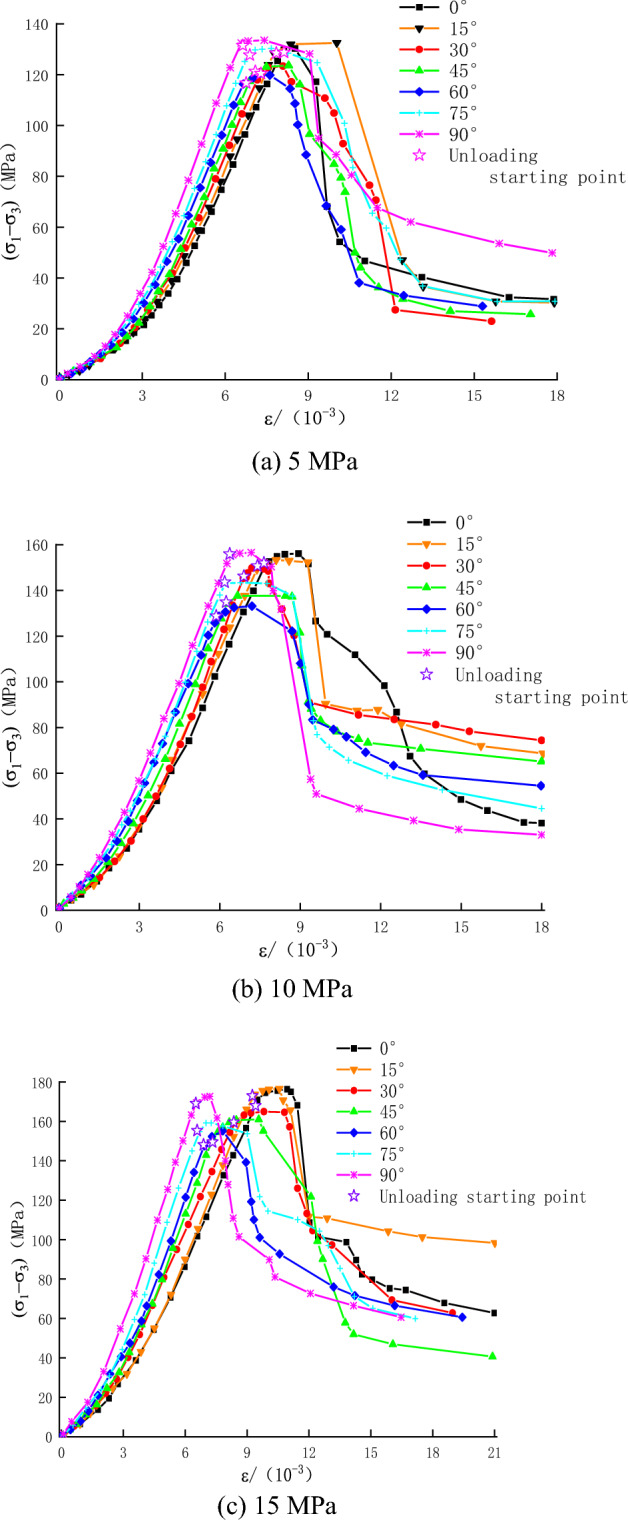

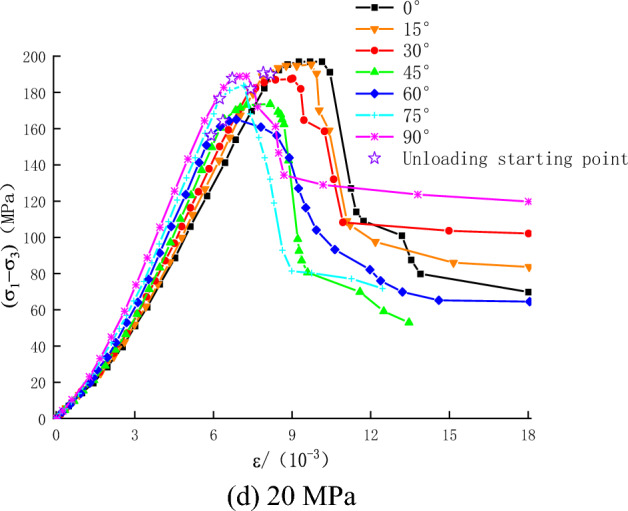
Stress–strain cures at different initial confining pressures.

The triaxial unloading stress–strain curves of the rock specimens with different bedding angles are presented in Fig. 5.

The following points can be noticed from Fig. 5:

- Generally, the stress–strain curves of rock specimens are similar at different bedding angles. In comparison, at the same initial confining pressure, as the bedding angle increased, the stress–strain curve became steeper (compaction phase), and the slope of the elastic phase gradually increased.
- During unloading of the confining pressure, the rock specimen reached a plastic state while the deviatoric stress increased. Relatively, the amount of plastic deformation was small at $\beta$ = 0° or 90°. During the unloading phase, the plastic deformation of the rock specimens was gradually increased when $\beta$ increased from 0° to 60° and $\beta$ decreased from 90° to 60°. The peak strength was initially decreasing and then increasing while $\beta$ increased.
- There was a significant difference in the stress–strain curve at the post-peak stage of rock specimens with different bedding angles. The axial strain increased slightly while the peak strength dropped to the residual strength (when $\beta$ = 0° or 90°), and the brittle failure characteristics were noticeable during the unloading stage. However, the brittleness characteristics were reduced while $\beta$ increased from 0° to 60° or decreased from 90° to 60°.

### Anisotropic deformation characteristics

**Figure 6:**
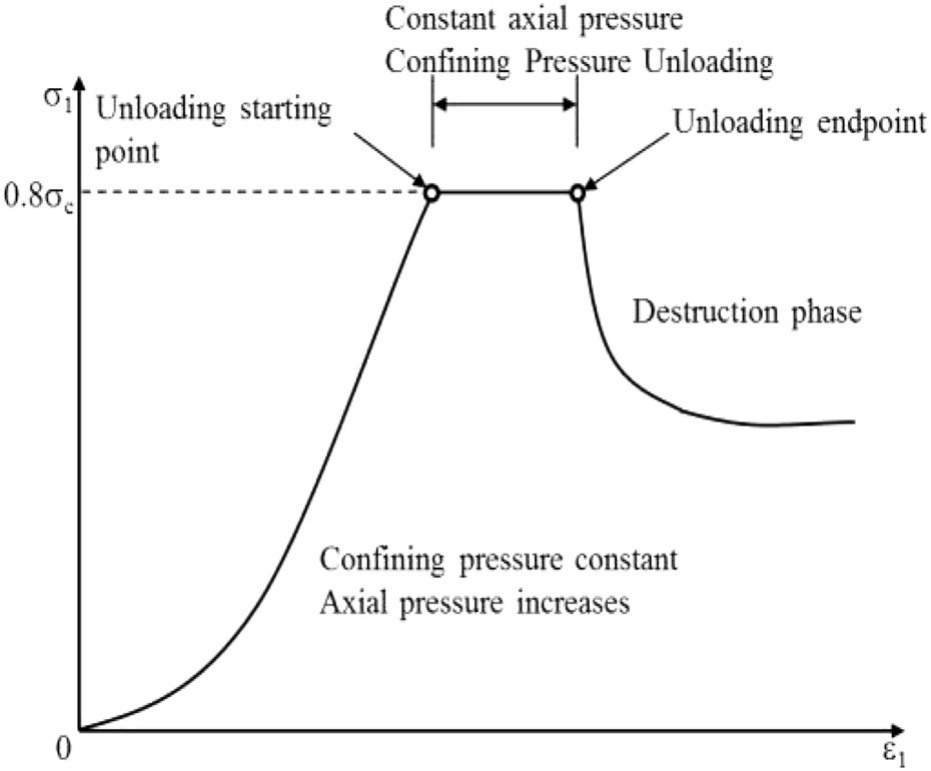
Typical stress–strain curve.

When the confining pressure was constant and the axial pressure was increased, anisotropic deformation characteristics were observed at different bedding angles. A similar phenomenon occurred while the axial pressure was constant and the confining pressure was unloaded (Fig. 6). Therefore, it is necessary to analyze the deformation characteristics of these two phases separately.

#### Deformation characteristics during the loading phase

**Figure 7:**
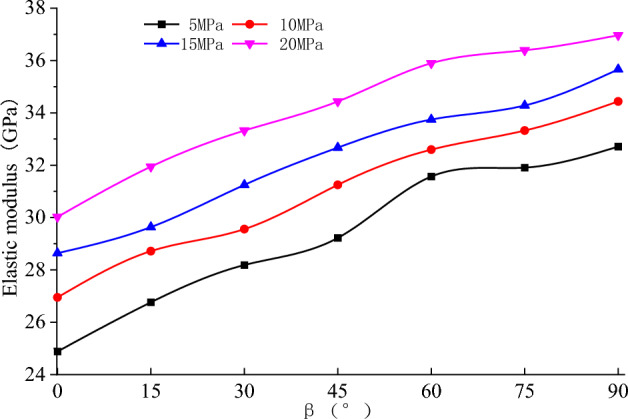
Relationship between elastic modulus and $\beta$ during loading.

**Figure 8:**
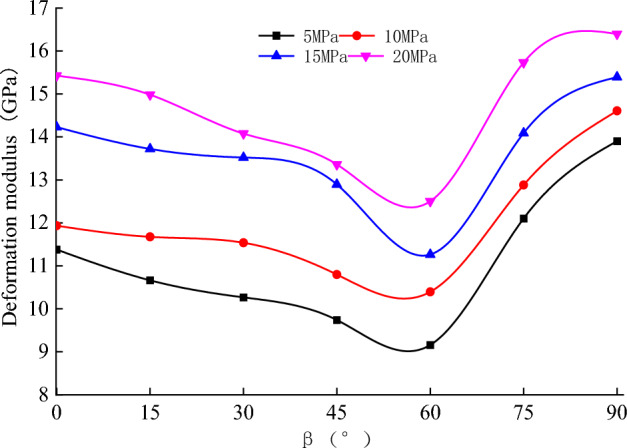
Relationship between the deformation modulus and $\beta$ during loading.

The relationships among elastic modulus, deformation modulus, and confining pressure and $\beta$ at the loading stage of sandstone are shown in Figs. 7 and 8. The elastic modulus takes the slope of the straight-line segment of the stress–strain curve, and the deformation modulus takes the Secant modulus corresponding to the compressive strength of 50%.

From Figs. 7 and 8, it can be seen that the bedding angle and confining pressure have a noticeable impact on the elastic modulus of bedded sandstone. The detailed descriptions are as follows.

The confining pressure has a compacting effect on the pores and fissures in a bedding plane and an inhibiting effect on micro-crack propagations. Therefore, the elastic modulus was increasing while the confining pressure increased. This has a good agreement with the previous studies^30–32^.

There was a gradual increase in the elastic modulus of rock specimens while the bedding angle increased (It illustrated a similar trend with Fig. 3). The elastic modulus increased rapidly when $\beta$ < 60° and increased slightly when $\beta$ > 60°. The reason for this is that the rock specimen is composed of the matrix and the bedding plane. In contrast, the stiffness of the rock matrix was higher than that of the bedding plane. At $\beta$ = 0°, the loading direction was perpendicular to the bedding plane, and the matrix and bedding plane were in series. The axial stiffness of the rock specimen is mainly governed by the bedding plane. Under the same axial stress increment, the compressive strain was relatively high, which resulted in a lower elastic modulus. However, when β = 90°, the reverse has occurred; because the loading direction was parallel to the bedding plane, the matrix and the bedding plane were parallel. The axial stiffness was governed by the rock matrix, and the responsible axial stress was relatively small, which yielded a higher elastic modulus. When the bedding angle increased from 0° to 90°, and the angle between the loading direction and the bedding plane changed from 90° to 0°, there was a gradual decrease in the axial strain under the same axial stress increment, which yielded a gradual increase in the elastic modulus.

The deformation modulus has a minimum and maximum value at $\beta$ = 60° and $\beta$ = 0° or 90°, respectively. Hence, the resulting graph was U-shaped. There are two reasons for this phenomenon. On the one hand, initially, the compressive strength was declined. Then, it was increasing while the bedding angle increased. Besides, 50% of the corresponding compressive strength was consistent. On the other hand, there was a short compressive section of the stress–strain curve at higher bedding angles (Fig. 5). In the meantime, the strain corresponding to 50% compressive strength decreased gradually. Under these two conditions, the deformation modulus was not consistent with the change in the elastic modulus.

#### Deformation characteristics during the unloading stage

The rate of axial strain variations was introduced to analyze the unloading condition of confining pressure on the axial strain increment ($\Delta {\dot{\varepsilon }}_{1}$)^36^. It is defined as the ratio of the axial strain increment to the confining pressure decrement that occurs between the initial point of unloading (the confining pressure) and the failure point:

1 $$\Delta {\dot{\varepsilon }}_{1}=\frac{\Delta {\varepsilon }_{1}}{\Delta {\sigma }_{3}}.$$

The rate of axial strain variations is the increment of the axial strain caused by the unit unloading of confining pressure. This variable reflects the effect of confining pressure unloading and rock deformation response on the axial deformation and state of stress, respectively. Unloading has a considerable impact on the deformation of rock at a higher rate of axial strain variations. During the unloading phase, anisotropic deformation characteristics of the bedded rock mass can be properly measured.

**Figure 9:**
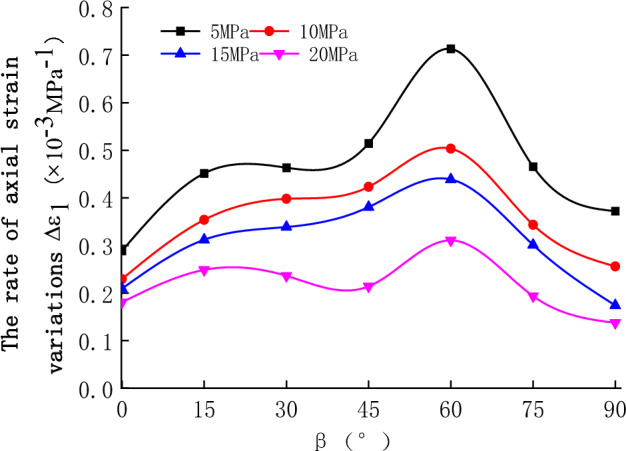
The relationship between the rate of axial strain variations and bedding angles at different initial confining pressures.

As can be seen from Fig. 9, the impact of the bedding angle and the confining pressure on the deformation of bedded sandstone at the unloading stage was considerable.

Initially, the rate of axial strain variations was increasing while the bedding angle increased. Later on, it had a decreasing trend. At $\beta$ = 0° or 90°, the rate of the axial strain variations was low. However, it has reached a peak value at $\beta$, approximately 60°. Between $\beta$ = 0° and 75°, the axial strain roughly exhibited an inverted U-shaped curve.

When the initial confining pressure was 5, 10, 15, or 20 MPa (at different bedding angles), the discrepancy between the minimum and maximum rates of the axial strain variations was 147%, 120%, 109%, and 72%, respectively. The impact of bedding angle on the rate of axial strain variations was decreasing while the confining pressure increased.

The anisotropy of rock specimens was noticeable during the unloading of the confining pressure, and it was mainly related to the deformation-failure characteristics of the rock specimens. The plastic deformation was low at the unloading phase (Fig. 7), and brittleness has decreased at $\beta$ = 0° or 90°. Similarly, the rate of axial strain variations was low. When $\beta$ increased from 0° to 60° or decreased from 90° to 60°, the plastic deformation of the rock specimen was gradually decreasing. Besides, the axial strain reached a peak value at $\beta$ approximately 60°. At the same time, the confining pressure inhibited the impact of the bedding plane. As a result, when the initial confining pressure was high (at different bedding angles), the discrepancy in the rate of axial strain variations had a gradual decrease.

### Anisotropic strength of bedded sandstones

#### Peak strength

**Figure 10:**
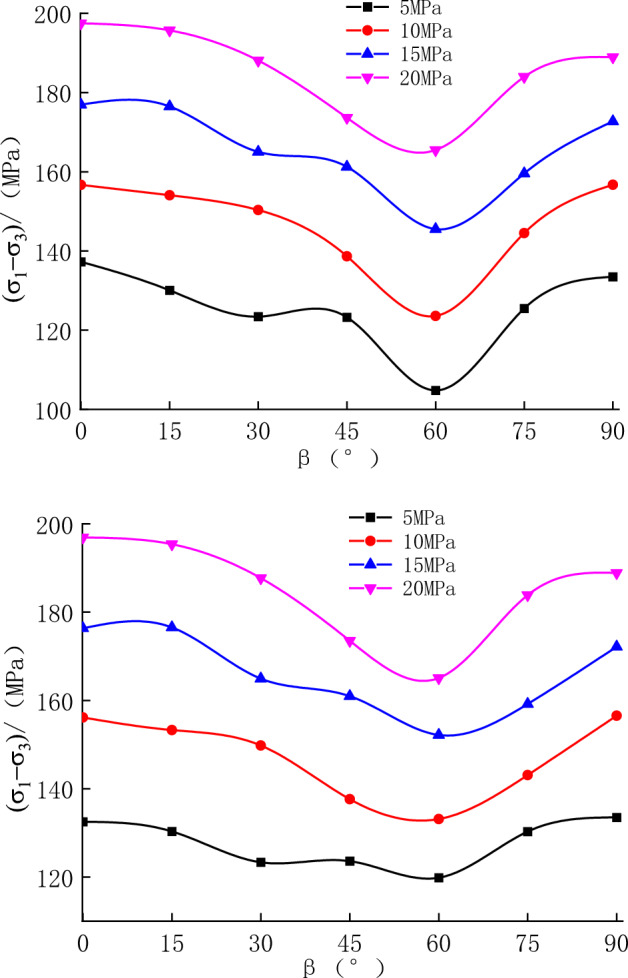
Relationship between peak strength and $\beta$ during unloading.

The relationships between the peak strength and the bedding angle are presented in Fig. 10. The compressive strength of the rock specimens was low when $\beta$ approximately 60° was. It reached a peak value at 0° and 90° and exhibited anisotropic characteristics. When the initial confining pressure values were 5, 10, 15, and 20 MPa, the difference between compressive strengths with different bedding angles were 31%, 27%, 22%, and 19%, respectively. In comparison, the impact of bedding angle on the compressive strength of rock specimens was gradually decreasing while the initial confining pressure increased.

#### Shear strength parameters

**Figure 11:**
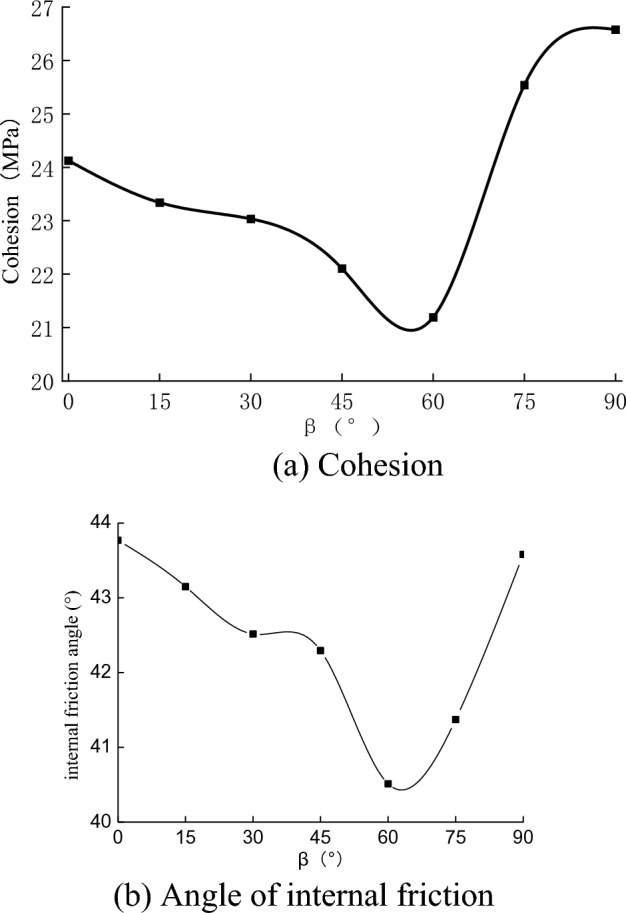
Relationship between cohesion, angle of internal friction, and bedding angle.

The strength of the rock specimens was evaluated based on the Mohr–Coulomb strength criterion. The cohesion and the angle of internal friction of the rock specimens at different bedding angles are shown in Fig. 11.

As shown in Fig. 11, there are obvious anisotropy of cohesion and internal friction angle at different bedding angles, and the graph was approximately U-shaped (0°–90°). The cohesion and the angle of internal friction of the rock specimens were relatively high at $\beta$ = 0° and 90°, and the cohesion and angle of internal friction of the rock specimens reached a small value at $\beta$ = 60°. The discrepancy between cohesion and angle of internal friction at different bedding angles was 20% and 7%, respectively.

## Discussion

### Anisotropic mechanical properties

The concept of an “anisotropic coefficient” was proposed by Singh^37^, to determine anisotropic rock strength quantitatively. It is the ratio between the maximum and minimum compressive strengths at bedding angle = 0° or 90°.

The mean anisotropy coefficient (${R}_{C}$) is defined as follows:

2 $${R}_{C}=\frac{{\sigma }_{c(\mathit{max})}}{{\sigma }_{c(\mathit{min})}},$$

where ${\sigma }_{c(\mathit{max})}$ is the maximum compressive strength at $\beta$ = 0° or 90° and ${\sigma }_{c(\mathit{min})}$ is the minimum compressive strength at $\beta$ = 0°–90°. A similar approach was used to calculate the anisotropic coefficients of the elastic modulus (*R*_E_), deformation modulus (*R*_Ea_) and compressive strength (*R*_C_) at the unloading failure of the rock specimens, as illustrated in Table 1.

**Table 1:** Anisotropy parameters of bedded sandstone (triaxial unloading).

Initial confining pressure (MPa)	Anisotropy coefficients		
	*R*_E_	*R*_Ea_	*R*_C_
5	1.31	1.51	1.31
10	1.27	1.41	1.27
15	1.24	1.36	1.22
20	1.22	1.31	1.19

The following points can be pointed out from Table 1.

The anisotropy parameters of the elastic modulus, deformation modulus, and compressive strength were mainly between 1.1 and 2.0. This range of anisotropy is regarded as low^31^.

The anisotropy coefficient of the rock specimens was also sensitive to the change in the confining pressure. As the initial confining pressure increased, the anisotropy coefficient showed a decreasing trend. When the initial confining pressure was 5 MPa, the anisotropy coefficients of the elastic modulus, deformation modulus, and compressive strength were 1.31, 1.51, and 1.31, respectively. Similarly, when the initial confining pressure was 20 MPa, the values decreased to 1.22, 1.31 and 1.19. The main reason for this is that the confining pressure has a compaction effect on the bedding plane and restrains the shear deformation and failure of the bedding planes. Hence, the anisotropy coefficients were reduced.

We compared the results of previous research^7^ with this paper, in order to more intuitively highlight the anisotropic characteristics of bedded sandstone under triaxial unloading. The main results of research^7^ were shown in Table 2.

**Table 2:** Anisotropy parameters of bedded sandstone (triaxial compression).

Initial confining pressure (MPa)	Anisotropy coefficients		
	*R*_E_	*R*_Ea_	*R*_C_
5	1.36	1.37	1.21
10	1.24	1.31	1.17
15	1.22	1.26	1.19
20	1.25	1.25	1.16

From Table 2, the following points can be pointed out.

The anisotropy parameters of the elastic modulus, deformation modulus, and compressive strength were mainly between 1.1 and 2.0. The conclusion is consistent with Table 1.

Generally speaking, the anisotropy coefficients of elastic modulus, deformation modulus and compressive strength of layered sandstone show similar regularities. The anisotropy of mechanical parameters of bedded sandstone decreases gradually with the increase of confining pressure. The anisotropy coefficients of elastic modulus, deformation modulus and compressive strength were 1.36, 1.37 and 1.21, respectively when the initial confining pressure was 5 MPa. When the initial confining pressure was 10 MPa, the anisotropy coefficients of elastic modulus, deformation modulus and compressive strength were decreased to 1.24, 1.31 and 1.17, respectively. Then the decreasing trend of anisotropy gradually slowed down, which generally belongs to low anisotropy^37^. When the confining pressure increased gradually, the influence of weak bedding plane on the anisotropy of compressive strength of rock samples decreased gradually, mainly because the confining pressure had a good compaction effect on the weak bedding plane. At the same time, it had a good lateral restraint effect on the shear deformation of weak bedding plane, and the anisotropy of elastic modulus, deformation modulus and compressive strength of bedded rock mass was weakened after the easy cracking of weak bedding plane was well restrained.

Through the comparison of Tables 1 and 2, it was found that the anisotropy coefficients of deformation modulus and compressive strength of bedded sandstone under triaxial unloading were larger than those under triaxial compression. Thus, it can be seen that the anisotropy of bedded sandstone is more obvious under the action of triaxial unloading. Based on this, the influence of bedding angle on the mechanical strength and deformation characteristics of rock mass cannot be ignored, which needs to be paid more attention in practical projects such as bedded rock slope excavation, tunnel design and deformation stability analysis.

### Anisotropic failure characteristics

**Figure 12:**
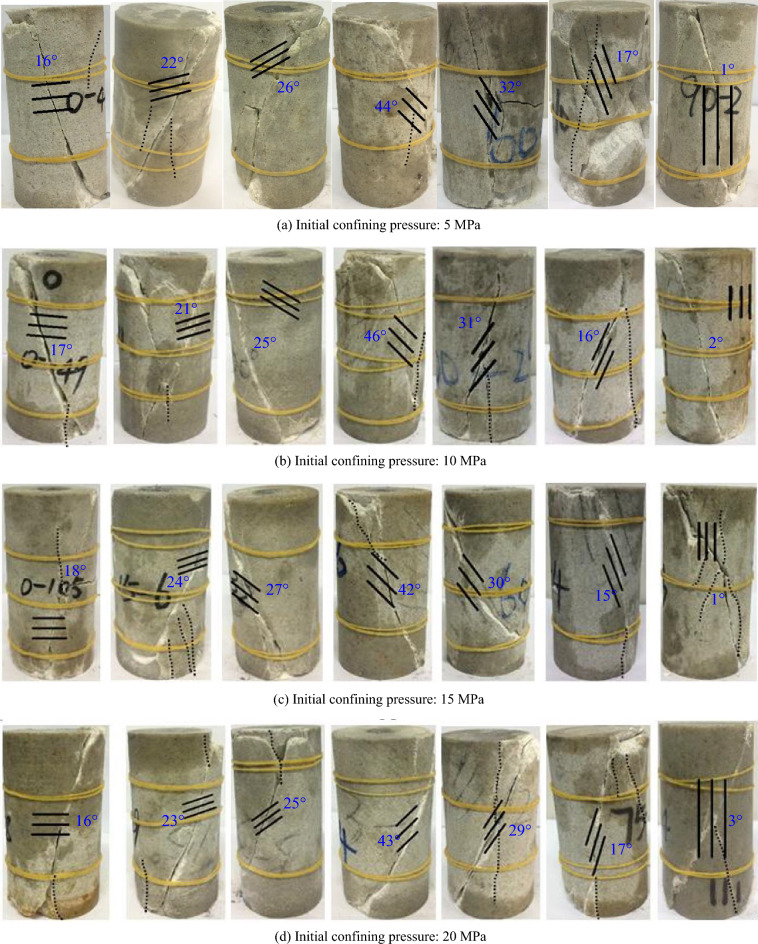
Typical cracked rock specimens.

The fractured of rock specimens at different bedding angles are shown in Fig. 12. The bedding angles are 0°, 15°, 30°, 45°, 60°, 75°, and 90° (from left to right).

The following points can be pointed out from Fig. 12:

- When $\beta$ = 0°, 15°, and 30°, the rupture plane intersected the rock matrix and the bedding plane, and the damage was generally a shear failure, and there were partial shear cracks near the two ends of the rock specimen. Moreover, there was consistency between the direction of the surface rupture and the bedding plane. This shows the bedding plane triggered the formation of surface rupture.
- When $\beta$ = 45°, the rock specimen developed shear cracks along the bedding plane during unloading. The meandering crack propagates to the tips of rock specimens under the influence of friction.
- When $\beta$ = 60° and 75°, the rupture surface of the rock specimen was generally in the direction of the bedding plane and was a typical shear-slip failure. Because of the unloading, there were some near-axial tension cracks on both sides of the shear fracture surface.

Axial tension cracks developed at the middle of the rock specimen along the bedding plane during unloading (when $\beta$ = 90°). The friction was responsible for the formation of large-angle shear cracks.

The anisotropy deformation and strength parameters were closely related to the failure morphology of rock specimens. Moreover, the variation in the rock morphology governed the rock deformation and strength.

After comprehensive analysis, the rock damage patterns were divided into four, as shown in Table 3.

**Table 3:** Unloading failure mode of bedded sandstone.

Item	Bedding angle	Failure model	Failure pattern	Compressive strength
1	0° ≤ *β* ≤ 30°		Through the matrix and bedding plane, skew shear damage	Higher
2	30° < *β* < 60°		Partial failure along the bedding plane/composite oblique shear failure passing through the matrix and bedding plane	Lower
3	60° ≤ *β* ≤ 75°		Shear-slip damage along the bedding plane, secondary tensile damage near the axis	Low
4	75° < *β* ≤ 90°		Partially along the bedding plane, local through the bedding plane, matrix composite tensile-shear damage	High

The dotted line in the figure shows the bedding plane, and the solid line shows the crack.

When 0° ≤ *β* ≤ 30°, there was a large angle between the major principal stress and the bedding plane. The failure mode was mainly governed by the stress state. Hence, a diagonal shear failure was observed across the matrix and bedding plane. The strength of the rock specimen was governed by the matrix and bedding plane. Accordingly, the corresponding strength was high.

When 30° < *β* < 60°, the angle between minor principal stress and the bedding plane gradually increased.

During unloading, the impact of bedding on the failure mode of the rock specimen has increased. The failure met the matrix and bedding plane. The strength of the rock specimen was governed by the matrix and the bedding plane. Hence, the corresponding strength was low.

When 60° ≤ *β* ≤ 75°, as calculated based on the Mohr–Coulomb strength criterion, the bedding and angle of shear failure angle were close to each other (45° + $\varphi$/2 = 67°), and hence the rock specimens were subjected to shear-slip failure along the bedding plane. Since the minor principal stress intersected a large bedding angle, the impact of unloading was noticeable, and axial tension cracks were developed on both sides of the shear fracture. The strength of the rock specimen was mainly governed by the bedding plane. Accordingly, the corresponding strength was low.

When 75° < *β* ≤ 90°, the major principal stress intersected a small bedding angle, and the impact of the bedding plane on the rock fracture pattern gradually diminished. Tension cracks were developed at the middle of the rock specimens, along the bedding plane during unloading. Due to the impact of friction on the rock specimen, a small angle was developed between the rupture and the bedding planes (on both ends), and composite shear failure was observed. The strength of the rock was governed by the rock matrix and the bedding plane. As a result, the corresponding strength was high.

In order to verify the rationality of the unloading failure mode of bedded sandstone proposed in this paper, the typical reservoir bank slopes in the Three Gorges reservoir area were selected for on-the-spot investigation, and the investigation results were compared with the failure mode proposed in this paper. The results were shown in Table 4.

**Table 4:** Comparison results of engineering cases.

Item	Bedding angle	Failure model	Failure pattern	Engineering case	Literature support
1	0° ≤ *β* ≤ 30°		Through the matrix and bedding plane, skew shear damage		Yin et al.^38^
2	30° < *β* < 60°		Partial failure along the bedding plane/composite oblique shear failure passing through the matrix and bedding plane		Zhao et al.^39^
3	60° ≤ *β* ≤ 75°		Shear-slip damage along the bedding plane, secondary tensile damage near the axis		Xu et al.^40^
4	75° < *β* ≤ 90°		Partially along the bedding plane, local through the bedding plane, matrix composite tensile-shear damage		Zhu et al.^41^

According to Table 4, it can be seen that the results of field investigation are highly consistent with the unloading failure mode of bedded sandstone proposed in this paper, and the bedding angle is the key factor leading to different failure patterns of reservoir bank slope. Similarly, we found that the unloading failure mode proposed in this paper can be well verified in Refs.^38–41^. Therefore, in the engineering slope, tunnel design and deformation stability analysis related to bedded rock mass, the influence of bedding angle on the mechanical properties and failure mode of rock mass cannot be ignored.

## Conclusions

The anisotropy of the bedded sandstone was noticeable. The elastic moduli of the rock specimens increased when the bedding angle increased. The deformation modulus, peak strength, cohesion, and angle of internal friction of the rock specimens exhibited a U-shaped trend. Initially, the shape was shrinking and then enlarging while the bedding angle increased. The rate of axial strain variations was increased and then decreased when the bedding angle increased during the unloading of the confining pressure. Most importantly, it has approximately an inverted U-shaped curve.

The confining pressure had a considerable weakening impact on the anisotropic mechanical properties of the bedded rock mass (during triaxial loading and unloading stages).

During triaxial unloading, the failure mode of the rock specimens was closely related to the bedding angle (which can be classified into four), and the anisotropy of the failure mode governed strength and deformation.

The bedding plane is a weak plane of the bedded sandstone. The anisotropy of unloading deformation, strength, and failure mode occurred due to weak cementation (sedimentary structure and bedding plane). When there was a large angle between the bedding plane and the unloading direction, shear failure was observed along the weak bedding plane.

## Data Availability

All data, models, and code generated or used during the study appear in the submitted article.
